# Factors Affecting Surgical Success Rates in Pediatric Horizontal Strabismus Surgery

**DOI:** 10.7759/cureus.74758

**Published:** 2024-11-29

**Authors:** Ali A Yetkin

**Affiliations:** 1 Department of Ophthalmology, Adiyaman University Faculty of Medicine, Adiyaman, TUR

**Keywords:** amblyopia, esotropia, exotropia, ocular motility disorders, strabismus, visual acuity

## Abstract

Introduction: Horizontal strabismus is a binocular alignment disorder that can lead to significant complications in childhood, such as visual impairment and amblyopia. This study aimed to evaluate the success rates of horizontal strabismus surgery in pediatric patients and to identify the factors affecting surgical outcomes. Specifically, it examined the relationship between surgical success and factors such as age, deviation angle, and amblyopia in pediatric horizontal strabismus cases.

Methods: The medical records of 95 patients diagnosed with horizontal strabismus and treated surgically between 2016 and 2022 were reviewed. Surgical success was defined by a postoperative deviation angle of less than 10 prism diopters (PD). Visual acuity and deviation angles were measured before and after surgery, and the data were analyzed statistically.

Results: Among the cases, 49 (51.5%) were diagnosed with esotropia and 46 (48.5%) with exotropia. One year after surgery, the overall success rate was recorded at 73.6%. The success rates were 77.6% in the esotropia group and 69.6% in the exotropia group, with no statistically significant difference between the groups (p=0.515). In age-based analyses, the surgical success rate was 73.8% in patients aged 0-9 years and 73.5% in those aged 10-18 years. Patients with a deviation angle greater than 45 PD had a success rate of 61.5%, while those with an angle below 45 PD achieved a success rate of 82.1% (p<0.05). Surgical success was observed in 50% of patients with amblyopia and 80.9% of those without amblyopia. Improvement in visual acuity was observed in 5.5% of non-amblyopic patients and in 22.7% of amblyopic patients; however, this improvement was not statistically correlated with surgical success.

Conclusions: This study revealed that factors such as the deviation angle and the presence of amblyopia affected the success rate of horizontal strabismus surgery. Surgical success rates did not differ significantly between esotropia and exotropia cases. Consistent with existing literature, patients with larger deviation angles and amblyopia were found to have lower surgical success rates.

## Introduction

Strabismus, a vision disorder characterized by a failure to achieve parallel alignment of the eyes, may result in critical issues such as diplopia, amblyopia, and loss of binocular vision. Strabismus is particularly significant in children, as visual development continues from infancy. It is essential to diagnose and initiate treatment in the early stages of childhood. If strabismus is not treated promptly, it may lead to permanent impairments in visual ability in the later stages of the child’s development. Furthermore, viewing strabismus as merely a cosmetic issue can negatively impact the social and psychological lives of affected children and their families [1,2]. Therefore, in addition to recognizing the clinical significance of this condition, it is crucial to consider its sociological and psychological dimensions. Studies conducted across various ethnic populations have reported that the global prevalence of strabismus in children ranges between 2% and 6% [3-5].

Childhood strabismus includes a variety of subtypes, with infantile esotropia, fully accommodative esotropia, partially accommodative esotropia, and intermittent exotropia being the most common. The prevalence of horizontal strabismus types, such as esotropia and exotropia, varies across different populations and age groups [6-8].

The current treatment options for strabismus include regular monitoring, medical interventions, and a variety of surgical procedures, including resection, recession, Faden operation, muscle transposition, marginal myotomy, disinsertion, myectomy, and graded recession [9,10]. The success rate of horizontal strabismus surgery is estimated to range from approximately 60% to 80%. Factors affecting surgical success include the age at onset of strabismus, duration before surgery, strabismus subtype, angle of deviation, and presence of amblyopia [11,12].

This study aimed to determine the surgical success rates in pediatric patients undergoing horizontal strabismus surgery and to identify factors affecting surgical success.

## Materials and methods

This study was designed retrospectively and conducted with the approval of the institutional ethics committee (date: November 15, 2022, decision number: 2022/8-21). A total of 95 patients (49 with esotropia and 46 with exotropia) who underwent surgery in our clinic between March 2016 and August 2022 were included. Written informed consent was obtained from the parents of all participants after explaining the study’s methodology and objectives in detail. The study was conducted in compliance with the principles of the Declaration of Helsinki. Patients with systemic diseases (such as diabetes mellitus and neurological conditions), those with a history of ocular surgery or trauma, cases of nerve palsy or restrictive strabismus, and those with nystagmus or Duane syndrome, as well as patients without regular follow-up, were excluded from the study.

For each patient included in the study, comprehensive preoperative and postoperative evaluations were performed. These included cycloplegic refraction measurement using 1% cyclopentolate (Cycloplegin®, Abdi Ibrahim, Istanbul, Turkey), applied three times at five-minute intervals and measured 45 minutes later; visual acuity assessed using Lea symbols, the “E” chart, and the Snellen chart; anterior segment examination via slit-lamp biomicroscopy; and dilated fundus examinations. Amblyopia was defined as a difference of two lines in best-corrected visual acuity (BCVA) values between the eyes or a BCVA value below 0.2 logMAR (0.63). Deviation angles at near (30 cm) and distance (6 m) fixation were recorded for patients using the Hirschberg light reflex test, cover-uncover test, alternate cover test, and prism test. These assessments measured deviations in primary and other positions across the nine cardinal gaze directions.

All operations were performed under general anesthesia by the same surgeon at the Ophthalmology Department of Adıyaman Training and Research Hospital. The surgical approach (bilateral recession, single muscle recession, or combined recession and resection on one eye) was determined based on the clinical characteristics of each patient’s strabismus. Postoperative success was defined as achieving a deviation angle of less than 10 prism diopters (PD) [13,14]. Follow-up evaluations were conducted preoperatively, at postoperative months 1, 3, and 6, and at one year after surgery.

Statistical analysis

Statistical analyses were performed using SPSS software, version 25.0 (IBM Corp., Armonk, NY). The Kolmogorov-Smirnov test was used to assess the normality of the data distribution. Categorical data were analyzed using the chi-square test, and numerical data were evaluated using the independent-samples t-test and Mann-Whitney U test. A p-value of less than 0.05 was considered statistically significant.

## Results

The study included a total of 95 patients (48 females and 47 males) diagnosed with strabismus. Among these, 49 patients had esotropia, and 46 had exotropia. The esotropia group included 26 females and 23 males, and the exotropia group consisted of 22 females and 24 males. The mean age at the diagnosis of esotropia was 3.69 ± 1.6 years, and the mean age at the time of surgery was 8.29 ± 1.1 years. The mean time from diagnosis to surgery in the esotropia group was 4.37 ± 1.3 years. In the exotropia group, the mean age at the time of strabismus diagnosis was 8.3 3 ± 2.9 years, the mean age at surgery was 15.43 ± 1.9 years, and the mean time from diagnosis to surgery was 6.17 ± 2.5 years. Table 1 summarizes the preoperative characteristics of patients by strabismus type, including age groups (0-9 and 10-18 years), mean refractive error, mean visual acuity, amblyopia status, mean anisometropia values and mean deviation angles.

**Table 1:** Preoperative clinical characteristics of patients with strabismus

	Esotropia	Exotropia	Total
Spherical values Right Left	2.34 ± 2.0 2.35 ± 2.0	-1.34 ± 3.1 -1.09 ± 2.3	0.55 ± 3.2 0.68 ± 2.7
Cylindrical values Right Left	-0.53 ± 1.0 -0.50 ± 2.0	-1.08 ± 1.6 -0.84 ± 1.1	-0.80 ± 1.3 -0.66 ± 1.2
Mean visual acuity Right Left	0.90 ± 0.2 0.90 ± 0.1	0.89 ± 0.2 0.96 ± 0.1	0.89 ± 0.2 0.94 ± 0.1
Anisometropia values Spherical Cylindrical	0.40 ± 0.8 -0.26 ± 0.4	-0.52 ± 1.4 -0.77 ± 1.3	-0.04 ± 1.2 -0.50 ± 1.0
0–9 age group	34 (35.8%)	8 (8.4%)	42 (44.2%)
10–18 age group	15 (15.8%)	38 (40.0%)	53 (55.8%)
Amblyopic	13 (13.7%)	9 (9.5%)	22 (23.2%)
Non-amblyopic	36 (37.9%)	37 (38.9%)	73 (76.8%)
Deviation angle	35.51 ± 10.3	41.02 ± 14.0	38.18 ± 12.5

Preoperative and postoperative deviation angles by strabismus type are shown in Table 2. Postoperative deviation angles were significantly reduced at all postoperative evaluations (p < 0.05).

**Table 2:** Preoperative and postoperative deviation angle measurements of patients with strabismus a: Month 1, b: Month 3, c: Month 6, d: Year 1, *p < 0.05 PD: prism diopter, SD: standard deviation

	Preoperative PD (Mean ± SD)	Postoperative PD (Mean ± SD)	p
Esotropia	35.51 ± 10.3	5.90 ± 5.7a	<0.001*
		7.08 ± 6.8b	<0.001*
		8.37 ± 7.5c	<0.001*
		10.08 ± 8.3d	<0.001*
Exotropia	41.02 ± 14.0	6.85 ± 6.8a	<0.001*
		8.43 ± 8.3b	<0.001*
		8.91 ± 9.7c	<0.001*
		9.93 ± 11.1d	<0.001*
Total	38.18 ± 12.5	6.36 ± 6.2a	<0.001*
		7.74 ± 7.6b	<0.001*
		8.63 ± 8.6c	<0.001*
		10.06 ± 9.7d	<0.001*

At the one-year follow-up, surgical success (postoperative deviation angle below 10 PD) was achieved in 70 of the 95 patients (73.6%), with 38 of the 49 esotropia cases (77.6%) and 32 of the 46 exotropia cases (69.6%) considered successful. No statistically significant difference in success rates was observed between the esotropia and exotropia groups (p = 0.515). Among the age groups, the success rate at one year was 73.8% (31/42) for those aged 0-9 years and 73.5% (39/53) for those aged 10-18 years (p = 0.833).

**Figure 1:**
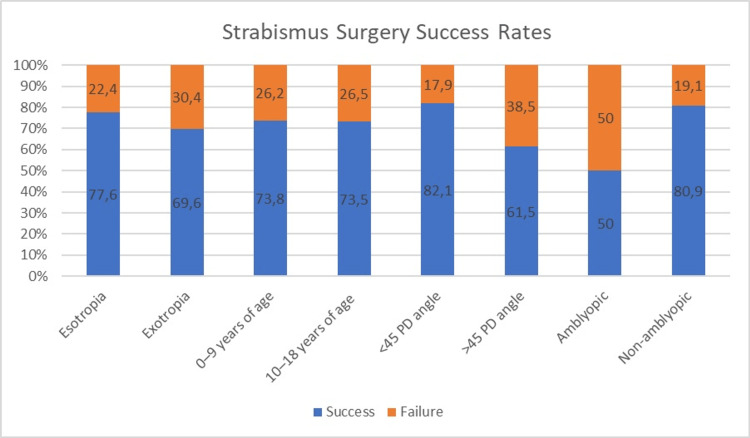
Factors affecting the success of strabismus surgery

Of the 56 patients with a preoperative PD value below 45, 46 (82.1%) achieved surgical success, while in those with a PD of 45 or higher, 24 of 39 (61.5%) presented with surgical success at one year. The difference between these groups was statistically significant (p < 0.05). Surgical success was achieved in 59 of the 73 non-amblyopic patients (80.9%) and in 11 of the 22 amblyopic patients (50%), with a significantly higher success rate in the non-amblyopic group (p < 0.05). The effects of various factors on surgical success are illustrated in Figure 1.

**Figure 2:**
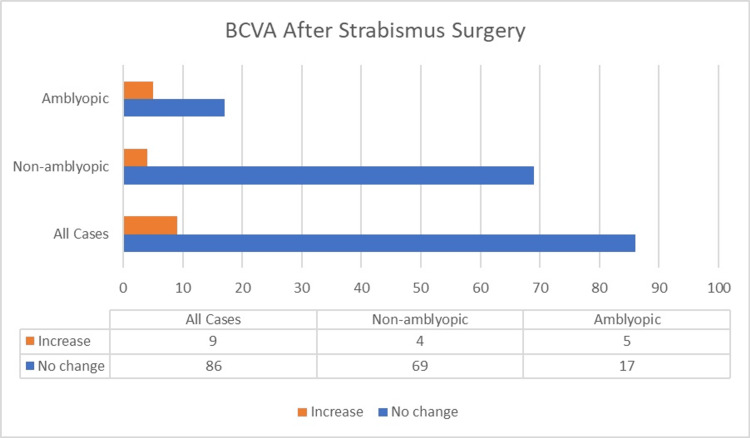
BCVA status of patients one year after strabismus surgery BCVA: Best-corrected visual acuity.

An increase in visual acuity was observed in four of the 73 non-amblyopic patients (5.5%) and in five of the 22 amblyopic patients (22.7%). The mean BCVA values before and one year after surgery were as follows: (0.993 ± 0.022 -0.975 ± 0.054) -(0.995 ± 0.025 -0.978 ± 0.053) (p = 0.321, p = 0.159) for non-amblyopic patients and (0.541 ± 0.248 -0.791 ± 0.270) -(0.568 ± 0.245 -0.814 ± 0.269) (p = 0,110, p = 0.096) for amblyopic patients. The one-year postoperative BCVA outcomes of the patients are presented in Figure 2.

## Discussion

Esotropia and exotropia, two types of horizontal strabismus, are common childhood conditions that can vary in prevalence based on race and age [15,16]. In our study of 95 patients, 49 (51.5%) had esotropia, while 46 (48.5%) had exotropia, with esotropia slightly more frequent among our participants.

Some of the studies examining the success of surgical procedures used in the treatment of horizontal strabismus have defined surgical success as a postoperative deviation angle of less than 20 PD, while others have set the threshold at less than 10 PD, reporting success rates of approximately 60% to 80% [11,17-20]. In our study, where we defined surgical success as a postoperative deviation angle of less than 10 PD, we found that 70 patients (73.6%) had surgical success at the one-year follow-up, which is consistent with the literature.

Studies assessing the effect of strabismus subtypes on horizontal strabismus surgery outcomes have indicated that postoperative success rates for esotropia and exotropia are nearly equivalent [12,13]. Similarly, in our study, we observed a surgical success rate of 77.6% in the esotropia group and 69.6% in the exotropia group. These results suggest no statistically significant difference in surgical success rates between the two groups.

Pandey et al. (2017) investigated factors affecting surgical outcomes for horizontal strabismus and found success rates of 41% in patients aged 0-9 years and 37% in older children, with no statistically significant difference [21]. Similarly, our study revealed success rates of 73.8% for the 0-9 age group and 73.5% for the 10-18 age group, also showing no significant difference between age groups.

In our cohort, surgical success was achieved in 82.1% of patients with a preoperative deviation angle of less than 45 PD, compared to 61.5% for those with a deviation of 45 PD or more. This finding aligns with previous studies reporting decreased surgical success with larger deviation angles [14,22,23]. The presence of amblyopia has been associated with reduced success in horizontal strabismus surgery [12,24]. In our study, success was achieved in 50% of amblyopic patients, compared to 80.9% in non-amblyopic patients, indicating a significantly lower success rate in amblyopic cases.

Waheeda-Azwa et al. reported that 91.8% of patients achieved a BCVA of 6/12 or better postoperatively, although this was not significantly correlated with surgical outcomes [25]. In our study, visual acuity improved in five amblyopic patients (22.7%) and in four non-amblyopic patients (5.5%). However, the mean BCVA values of the two groups at one year after surgery did not differ significantly.

Our study has several limitations. First, due to its retrospective design, there were challenges in controlling certain variables and accessing patient history during the data collection process. In a retrospective study design, difficulties in controlling certain variables may arise because data may have been collected using different protocols, standards, or measurement techniques. These differences may lead to inconsistencies in the data collection process. Second, the limited number of patients included in the study reduces the generalizability of the findings; therefore, further studies with a larger number of participants are needed to adapt these results to a broader population. Lastly, the relatively short follow-up period limits our ability to evaluate the long-term sustainability of surgical success and potential late-onset complications. In this context, we believe that prospective studies conducted across various healthcare centers involving larger patient groups and extended follow-up periods could provide more comprehensive and reliable data on the outcomes of horizontal strabismus surgery for the general population. Such advanced research could contribute to the development of treatment protocols and enhance the long-term sustainability of surgical success.

## Conclusions

In our study, factors affecting surgical success in horizontal strabismus among pediatric patients were identified as larger deviation angles and the presence of amblyopia. Although the results also indicated that the presence of amblyopia reduced the surgical success rate, there was an increase in visual acuity in amblyopic patients compared to those without amblyopia. Nevertheless, this increase did not show a statistically significant difference. In our study, we found that the surgical success rates for strabismus were higher in patients without amblyopia. Therefore, it would be more appropriate to perform strabismus surgery after completing amblyopia treatment in patients with amblyopia.
